# 4-aminopyridine reverses ataxia and cerebellar firing deficiency in a mouse model of spinocerebellar ataxia type 6

**DOI:** 10.1038/srep29489

**Published:** 2016-07-06

**Authors:** Sriram Jayabal, Hui Ho Vanessa Chang, Kathleen E. Cullen, Alanna J. Watt

**Affiliations:** 1 Department of Biology, McGill University, Montreal, H3G 0B1, Canada; 2 Integrated Program in Neuroscience, McGill University, Montreal, H3G 0B1, Canada; 3 Department of Physiology, McGill University, Montreal, H3G 1Y6, Canada

## Abstract

Spinocerebellar ataxia type 6 (SCA6) is a devastating midlife-onset autosomal dominant motor control disease with no known treatment. Using a hyper-expanded polyglutamine (84Q) knock-in mouse, we found that cerebellar Purkinje cell firing precision was degraded in heterozygous (SCA6^84Q/+^) mice at 19 months when motor deficits are observed. Similar alterations in firing precision and motor control were observed at disease onset at 7 months in homozygous (SCA6^84Q/84Q^) mice, as well as a reduction in firing rate. We further found that chronic administration of the FDA-approved drug 4-aminopyridine (4-AP), which targets potassium channels, alleviated motor coordination deficits and restored cerebellar Purkinje cell firing precision to wildtype (WT) levels in SCA6^84Q/84Q^ mice both in acute slices and *in vivo*. These results provide a novel therapeutic approach for treating ataxic symptoms associated with SCA6.

Spinocerebellar ataxias (SCAs) are a group of rare diseases arising from diverse genetic etiology that share common symptoms. SCA6 is an autosomal dominant disease affecting ~5 per 100,000 people worldwide^1^ that is characterized by progressive ataxia, dysarthria, and nystagmus^1^. Currently, there is no treatment available for this devastating disease^1^. SCA6 is caused by the expansion of a CAG triplet repeat in the *CACNA1A* gene encoding the α1A subunit of the P/Q type voltage-gated Ca^2+^ channel (P/Q channel), and a C-terminus fragment, α1ACT, resulting in a polyglutamine (polyQ) expansion^2^. P/Q channels are highly expressed in cerebellar Purkinje cells^3^,^4^, and postmortem studies demonstrate Purkinje cell degeneration in SCA6 patients^5^. To date, several mouse models of SCA6 have been developed^6^,^7^,^8^,^9^ that display hallmark features of SCA6, including progressive motor coordination deterioration, cellular inclusions in Purkinje cells, and Purkinje cell degeneration. Mouse models fall broadly into two classes: (1) knock-in models with hyper-expanded polyQ repeats^6^,^7^, or (2) models with overexpression of pathological C-terminus fragments of the α1A subunit of the P/Q channel^8^,^9^. The latter models produces similar motor coordination abnormalities as hyper-expanded models, and thus may underlie part or all of the ataxic phenotype of SCA6. In the present study, we used a hyperexpanded (84Q) mouse model of SCA6^6^ that displays late-onset motor deficits in both heterozygous (SCA6^84Q/+^) mice at 19 months^6^, and homozygous (SCA6^84Q/84Q^) mice at 7 months^6^,^10^, similar to human patients (e.g.^11^). Purkinje cell loss is observed at 2 years in homozygous SCA6^84Q/84Q^ mice^10^, suggesting that altered Purkinje cell function may contribute to SCA6 onset at 7 months prior to cell death.

Purkinje cells are the sole output neurons of the cerebellar cortical microcircuit, and are thus ideally poised to influence cerebellar-related function. While Purkinje cell firing output is shaped by synaptic input, Purkinje cells fire action potentials in the absence of synaptic drive at characteristic frequencies ranging from 20–200 Hz, with high precision in the timing of their action potentials, giving Purkinje cell pacemaker-like properties^12^,^13^,^14^. P/Q channels are known to contribute to spontaneous Purkinje cell firing properties^15^, and several P/Q channel mutations^16^,^17^ display reduced Purkinje cell firing precision and ataxia. In fact, changes in Purkinje cell firing rate and/or firing precision are cellular deficits that have often been associated with ataxia: for example, in episodic ataxia type 2 (EA2)^16^,^18^,^19^, SCA1^20^,^21^,^22^, SCA2^23^,^24^, SCA3^25^, as well as in late disease stages in an α1ACT overexpression mouse model of SCA6^9^.

To address the role of Purkinje cell firing in SCA6, we studied Purkinje cell firing properties from heterozygous and homozygous SCA6^84Q^ mice at ages when motor coordination deficits were observed. We found that alterations in Purkinje cell firing precision can be detected in heterozygous mice at 19 months, when motor deficits were also observed^6^. Similar alterations in firing precision were seen in homozygous mice at 7 months, when motor deficits were observed in these mice^6^,^10^, demonstrating common pathophysiology for homozygous and heterozygous SCA6^84Q^ mice in a gene dose-age dependent manner. Next, we found that changes in firing output could be rescued acutely with the drug 4-aminopyridine (4-AP) that has similarly rescued firing precision alterations in a different form of ataxia, EA2^18^, both *in vitro* and *in vivo*. Finally, we show that chronic oral administration of 4-AP rescues ataxic symptoms in SCA6^84Q/84Q^ mice, and that this rescue is associated with a recovery of Purkinje cell firing precision. These findings suggest a first pharmacological approach for treating SCA6.

## Results

### Loss of Purkinje cell firing precision in both heterozygous and homozygous SCA6^84Q^ mice

**Figure 1:**
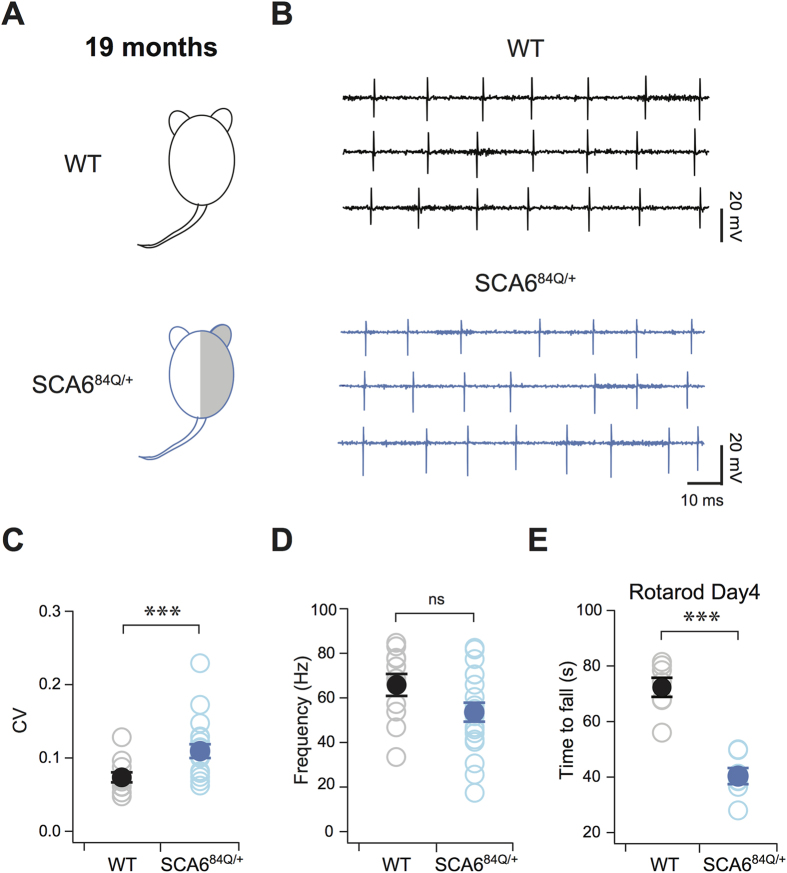
Firing precision deficits in heterozygous SCA6^84Q/+^ mice. (**A**) 19-month-old heterozygous WT and SCA6^84Q/+^ mice. (**B**) Sample traces from a Purkinje cell from WT (black, top) and SCA6^84Q/+^ mice (blue, bottom) illustrate the loss of Purkinje cell firing precision at 19 months. (**C**) Purkinje cell firing precision, as measured by the coefficient of variation (CV) of inter-spike intervals of Purkinje cell action potentials, is reduced in 19-month-old heterozygous SCA6^84Q/+^ mice (WT: CV = 0.08 ± 0.005; SCA6^84Q/+^: CV = 0. 11 ± 0.010; P = 0.0047). (**D**) However, no changes in firing frequency was observed in 19-month-old SCA6^84Q/+^ mice (WT: frequency = 65.8 ± 4.9 Hz, SCA6^84Q/84Q^: frequency = 53.6 ± 4.23 Hz, P = 0.071; N = 11 WT and N = 19 SCA6^84Q/+^ for (**C**,**D**)). (**E**) Motor coordination deficit was observed in SCA6^84Q/+^ mice at 19 months (WT mice spent 78.7 ± 10.3 s on Rotarod before falling on day 4, N = 7, grey/black markers; SCA6^84Q/84Q^ mice spend 26.3 ± 4.2 s on Rotarod on day 4, N = 7, light/dark blue markers; P = 0.0016). Comparisons made with Student’s t test; ***P < 0.005.

Using a mouse model of SCA6 harboring a humanized *CACNA1A* gene encoding a hyper-expanded (84Q) repeat^6^, we explored the cellular pathophysiology underlying the onset of ataxia. Most human SCA6 cases are autosomal dominant^1^, meaning that only one copy of the expanded gene is necessary to produce disease symptoms. Accordingly, to understand the cerebellar pathophysiology underlying loss of motor coordination, we first tested heterozygous mice at 19 months that have previously been described to display motor coordination deficits^6^ as a model for human SCA6. Non-invasive extracellular recordings were made to measure the spontaneously firing Purkinje cells in acute cerebellar slices from 19-month-old litter-matched WT and heterozygous SCA6^84Q/+^ mice (Fig. 1A). WT Purkinje cells fired action potentials with highly precise timing at 19 months, reflected in low values of the coefficient of variation (CV) of the inter-spike intervals (Fig. 1B top; 1C). We found that the precision of action potential timing was significantly reduced in heterozygous SCA6^84Q/+^ Purkinje cells compared to litter- and age-matched WT neurons (P = 0.0047; Fig. 1B bottom; 1C). The range of firing frequencies observed in SCA6^84Q/+^ Purkinje cells (ranging from 17–83 Hz) was similar to WT mice at 19 months (ranging from 33–85 Hz) and there were no significant differences observed in firing frequency (P = 0.71; Fig. 1D). Using an accelerating Rotarod assay, we confirmed that motor coordination was impaired in SCA6^84Q/+^ mice at 19 months compared to WT (P = 0.0016; Fig. 1E) in agreement with previous studies^6^. These results suggest that changes in the precision of action potential timing occur in SCA6 mice when motor coordination deficits are present.

**Figure 2:**
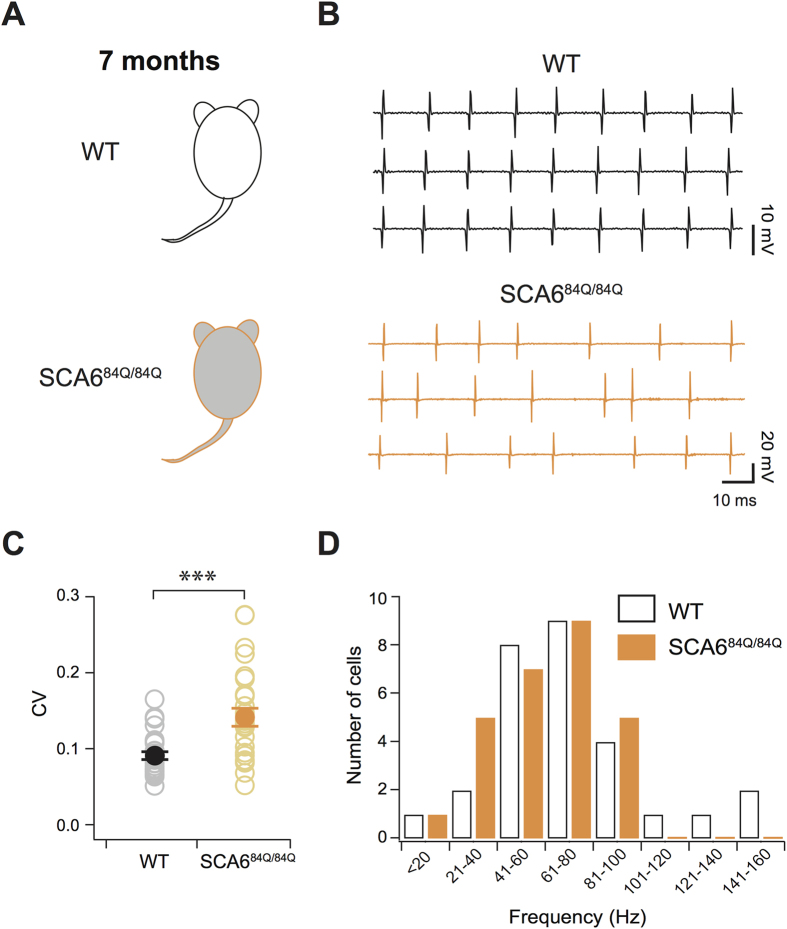
Firing precision and rate deficits in homozygous SCA6^84Q/84Q^ mice. (**A**) 7-month-old homozygous WT and SCA6^84Q/84Q^ mice. (**B**) Sample traces show action potentials from a single Purkinje cell from a WT (black, top) and an SCA6^84Q/84Q^ (orange, bottom) mouse at 7 months. (**C**) Changes in firing precision demonstrated by an elevated CV of inter-spike intervals for Purkinje cell action potential firing in SCA6^84Q/84Q^ mice (WT: CV = 0.09 ± 0.005; SCA6^84Q/84Q^: CV = 0.14 ± 0.01; P < 0.0001). (**D**) Histogram showing firing rates of individual Purkinje cells from SCA6^84Q/84Q^ mice (orange) and WT Purkinje cells (black); note the absence of Purkinje cells firing at frequencies >100 Hz in SCA6^84Q/84Q^ mice. Average WT Purkinje cell firing rate = 71.7 ± 6.1 Hz; average SCA6^84Q/84Q^ Purkinje cell firing rate = 56.4 ± 3.9 Hz; P = 0.041; N = 28 for WT, N = 26 for SCA6^84Q/84Q^ for (**C**,**D**). Comparisons made with Student’s t tests; ***P < 0.005.

Although heterozygous SCA6^84Q/+^ mice are a good model system for SCA6, it is impractical to conduct experiments routinely at 19 months in aged mice. We then turned to homozygous SCA6^84Q/84Q^ mice, which show motor coordination deficits at 7 months^6^,^10^ to determine whether similar changes in firing precision are observed in homozygous mice as in heterozygotes. We recorded spontaneous Purkinje cell action potentials in cerebellar slices from SCA6^84Q/84Q^ and WT neurons at 7 months (Fig. 2A). As at 19 months, we found that WT Purkinje cells fired action potentials with highly precise timing at 7 months, reflected in low CV (Fig. 2B top, 2C); and the range of firing frequency across WT Purkinje cells was broad (ranging from 15–147 Hz; Fig. 2D), as previously reported^13^,^14^. Homozygous SCA6^84Q/84Q^ mice displayed reduced precision in the timing of Purkinje cell action potentials, reflected in higher CV of inter-spike intervals (P < 0.0001; Fig. 2B bottom, 2C), and fired action potentials over a significantly narrower range of firing frequencies (19–91 Hz) than WT Purkinje cells (P = 0.041; Fig. 2D). Since Purkinje cell firing precision was altered in both heterozygous SCA6^84Q/+^ and homozygous SCA6^84Q/84Q^ mice at the ages when motor deficits emerge (19 and 7 months, respectively), while changes in firing rate were only observed in homozygous SCA6^84Q/84Q^ mice, our results provide support for the use of homozygous SCA6^84Q/84Q^ mice at 7 months as a mouse model of SCA6.

### 4-AP acutely rescues Purkinje cell firing precision in SCA6^84Q/84Q^ mice

**Figure 3:**
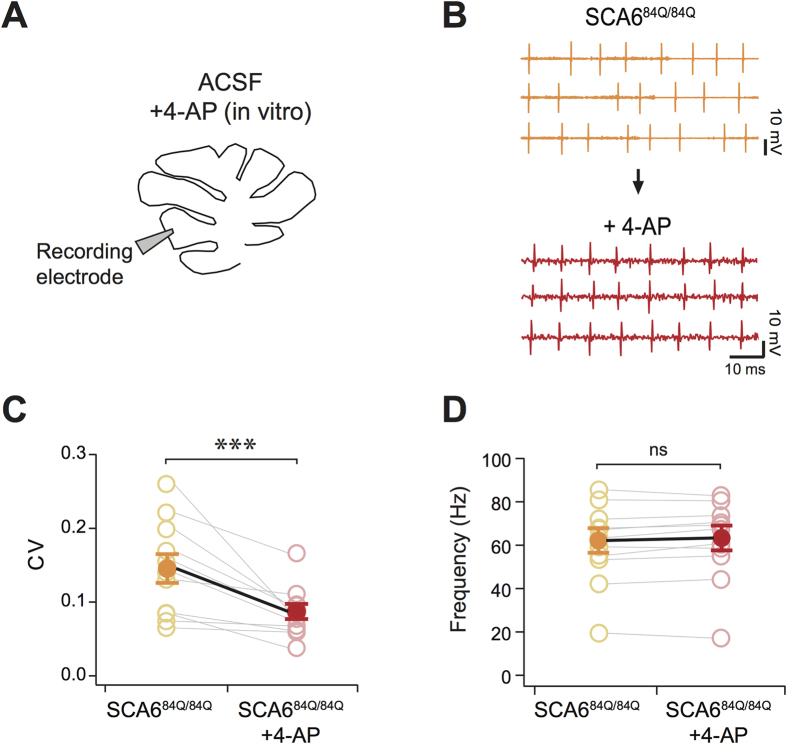
Acute 4-AP rescues firing precision deficits in SCA6^84Q/84Q^ mice. (**A**) Acute application of 5 μM 4-AP in cerebellar slices from SCA6^84Q/84Q^ and WT mice. (**B**) Representative traces from SCA6^84Q/84Q^ mouse Purkinje cell before (orange, top) and after (red, bottom) 4-AP wash-in. (**C**) Purkinje cell spiking becomes more regular with the application of 4-AP (CV was reduced after 4-AP application, CV_before_ = 0.15 ± 0.02; CV_after_ = 0.10 ± 0.009; paired Student’s t test, P = 0.0027; N = 11), while (**D**) not altering firing rate (frequency_before_ = 60.7 ± 5.6 Hz; frequency_after_ = 61.8 ± 5.6 Hz; P = 0.15). ***P < 0.005.

Purkinje cell action potential pacemaker properties have been linked to K^+^ channel function^16^,^18^,^19^, so we hypothesized that the FDA-approved K^+^ channel blocker drug 4-aminopyridine (4-AP), that is used in patients to treat Multiple Sclerosis (MS)^26^,^27^, would influence the precision timing of Purkinje cell action potentials in SCA6. To test this, we acutely applied 4-AP on cerebellar slices prepared from 7 month SCA6^84Q/84Q^ mice (Fig. 3A). We found that 4-AP significantly increased the precision of action potentials in Purkinje cells from 7-month-old SCA6^84Q/84Q^ mice (P = 0.0027; Fig. 3B,C) to levels indistinguishable from WT control Purkinje cell action potential precision (F_2, 68_ = 8.55, P = 0.90), while not significantly changing Purkinje cell firing rates (P = 0.15; Fig. 3B,D). To explore whether this effect was due to 4-AP acting on presynaptic K^+^ channels affecting presynaptic action potentials and/or presynaptic release^28^,^29^, we applied 4-AP after the addition of a cocktail of blockers of fast excitatory (AMPA and NMDA) and inhibitory (GABA_A_) synaptic transmission, and found that the reduction of firing precision in SCA6^84Q/84Q^ Purkinje cells caused by 4-AP was not occluded with presynaptic neurotransmitter blockade (4-AP reduces CV after synaptic blockade, P = 0.0008; magnitude of change is not different from without synaptic blockade, P = 0.43; Supplementary Fig. 1). Purkinje cell firing rates were increased by the application of synaptic blockers, likely due to the removal of GABA-mediated tonic inhibition^13^ (P < 0.0001; Supplementary Fig. 1). These results argue that 4-AP recovers Purkinje cell firing precision by its action on intrinsic Purkinje cell channels^18^ rather than on channels located on presynaptic neurons^29^ in our mouse model of SCA6.

### Chronic 4-AP improves motor coordination in SCA6^84Q/84Q^ mice

**Figure 4:**
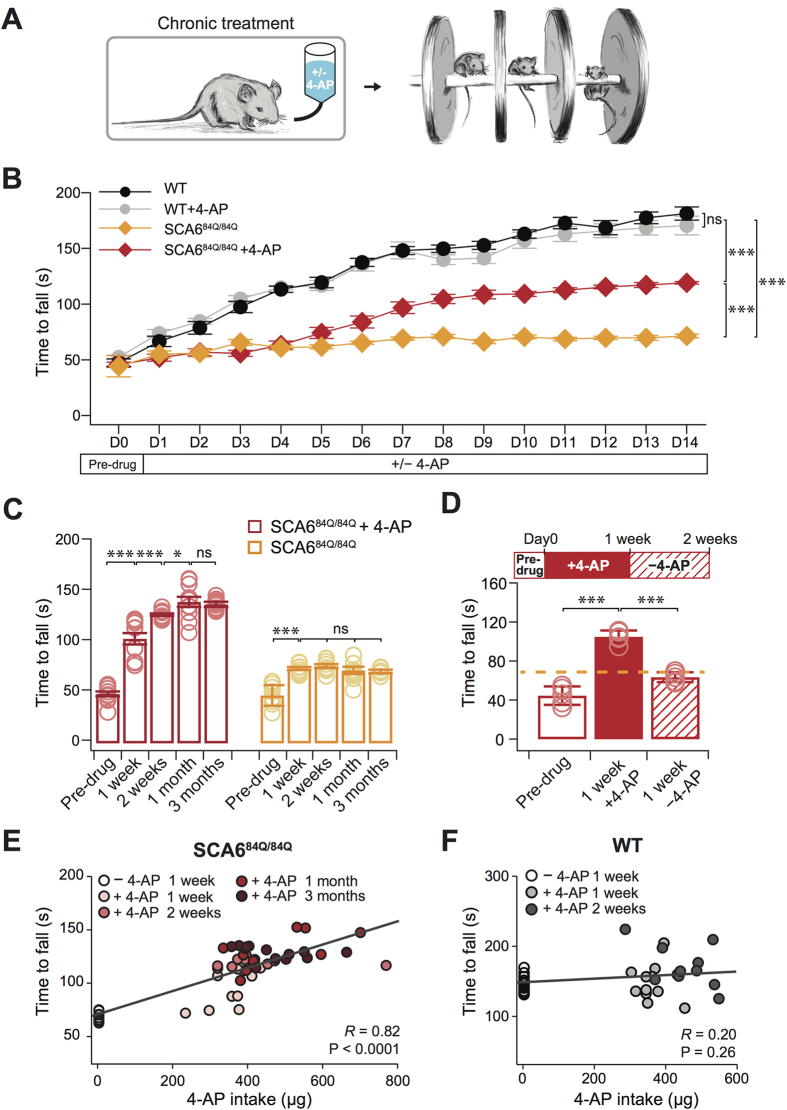
Chronic orally administered 4-AP improves motor coordination in SCA6^84Q/84Q^ mice. (**A**) Experimental set-up. (**B**) Rotarod performance was reduced in SCA6^84Q/84Q^ mice (orange) compared to litter-matched WT mice (black; P < 0.0001). Chronic 4-AP administration improved the motor coordination in SCA6^84Q/84Q^ mice (SCA6^84Q/84Q^ + 4-AP, red; different from SCA6^84Q/84Q^ with vehicle, orange; P < 0.0001), although rescue was incomplete to WT levels (P < 0.0001). Oral administration of 4-AP caused no change in WT mice performance (grey; P = 0.99; ANOVA followed by post-hoc Tukey test for all comparisons; N = 11 for WT with vehicle; N = 11 for WT + 4-AP; N = 9 for SCA6^84Q/84Q^ mice with vehicle; N = 11 for SCA6^84Q/84Q^ + 4-AP). (**C**) Chronic 4-AP administration improved motor coordination after 1 week (red bars: Pre-drug versus 1 week 4-AP, P < 0.0001), continued to improve after 2 weeks, (1 versus 2 weeks 4-AP, P = 0.0024) and showed further improvement after 1 month of 4-AP (2 weeks versus 1 month 4-AP, P = 0.02; N = 11 SCA6^84Q/84Q^ mice); motor performance remained elevated for as long as 3 months (1 versus 3 months not different, P = 0.45; ANOVA followed by post-hoc Tukey test). Motor performance improved in SCA6^84Q/84Q^ mice without drug after 1 week because of motor learning (Pre-trial versus 1 week no drug, P < 0.0001), and then plateaued (1 versus 2 weeks, P = 0.20; 2 weeks versus 1 month, P = 0.27; 1 versus 3 months, P = 0.84). (**D**) Improved performance requires the continual administration of 4-AP, as withdrawal of 4-AP resulted in declining Rotarod performance levels (1 week − 4-AP after 1 week + 4-AP, versus 1 week + 4-AP, P < 0.0001; significantly different; N = 5 SCA6^84Q/84Q^ mice; paired Student’s t test, Bonferroni corrected, α = 0.025). Dashed orange line shows 2 week SCA6^84Q/84Q^ mice – 4-AP. (**E**) There was a strong correlation between motor performance and drug intake in SCA6^84Q/84Q^ mice, while (**F**), the performance of WT mice was not correlated to drug intake (±4-AP). *P < 0.05, ***P < 0.005.

Since 4-AP rescues the Purkinje cell firing precision deficit that we hypothesize may underlie the onset of ataxia in our mouse model of SCA6, we wondered whether it might also ameliorate ataxia. To examine this, we used oral administration to chronically deliver 4-AP or vehicle (10% sucrose water) to 7-month-old homozygous SCA6^84Q/84Q^ and WT mice (Fig. 4A left)^18^, and assayed ataxic symptoms using an accelerating Rotarod assay, which we have previously shown is a robust assay for detecting motor coordination deficits in SCA6^84Q/84Q^ mice (Fig. 4A right)^10^. We found that homozygous SCA6^84Q/84Q^ mice given 4-AP showed improved motor coordination compared to SCA6^84Q/84Q^ given vehicle (P < 0.0001; Fig. 4B), while WT mice exposed to 14 days of 4-AP had motor performance indistinguishable from WT with vehicle (P = 0.99; Fig. 4B). We examined several different time points following 4-AP administration and found that motor performance improved in SCA6^84Q/84Q^ mice after 1 week (Fig. 4C), and continued to improve after 2 weeks and 1 month of 4-AP administration, whereupon it remained significantly elevated up to 3 months of administration (Fig. 4C). To further confirm that the improvement of motor performance depended on 4-AP, we withdrew 4-AP after 1 week of administration, and observed that the Rotarod performance of SCA6^84Q/84Q^ mice decreased to control levels after 1 week of 4-AP withdrawal (Fig. 4D). Interestingly, there was a significant positive correlation between Rotarod performance and drug consumption in SCA6^84Q/84Q^ mice but not in WT mice (SCA6^84Q/84Q^ mice: *R* = 0.82, P < 0.0001; WT mice: *R* = 0.20, P = 0.26; Fig. 4E,F), suggesting a dose-response relationship of 4-AP on motor coordination in SCA6^84Q/84Q^ mice.

### Chronic 4-AP rescues Purkinje cell firing deficit in SCA6^84Q/84Q^ mice

**Figure 5:**
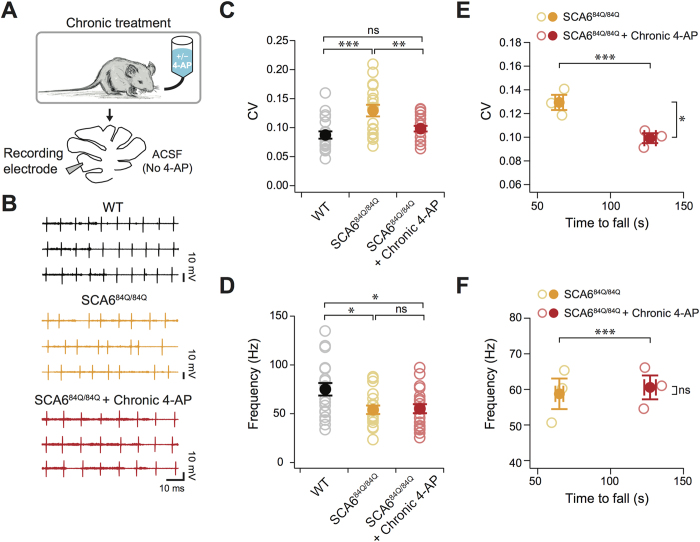
Chronic 4-AP restores *in vitro* Purkinje cell firing precision in SCA6^84Q/84Q^ mice. (**A**) After chronic 4-AP or vehicle treatment for 3 months, Purkinje cell action potentials were recorded in the absence of 4-AP in the extracellular solution in acute slices. (**B**) Sample recordings from age-matched WT (top, black traces), vehicle-treated SCA6^84Q/84Q^ (middle, orange traces), and chronic 4-AP-treated SCA6^84Q/84Q^ mice (bottom, red traces) showing that the firing precision is recovered in 4-AP-treated mice. (**C**) Spike precision, as measured by the CV of inter-spike intervals from Purkinje cell spike trains from WT, vehicle-treated SCA6^84Q/84Q^, and 4-AP-treated SCA6^84Q/84Q^ mice, shows a significant reduction after 4-AP treatment to levels that are indistinguishable from WT (WT: CV = 0.09 ± 0.01; SCA6^84Q/84Q^ with vehicle: CV = 0.13 ± 0.01; SCA6^84Q/84Q^ + 4-AP: CV = 0.10 ± 0.01). (**D**) Firing frequency is however unaffected by 4-AP treatment in SCA6^84Q/84Q^ mice, and both show significant reduction from WT levels (WT: frequency = 76.6 ± 5.5 Hz; SCA6^84Q/84Q^ with vehicle: frequency = 58.8 ± 3.7 Hz; SCA6^84Q/84Q^ + 4-AP: frequency = 59.7 ± 3.9 Hz). N = 20 for WT, N = 18 for untreated SCA6^84Q/84Q^, and N = 20 for SCA6^84Q/84Q^ + 4-AP for (**C**,**D**). (**E**,**F**) For individual animals, 4-AP treatment produced a significant increase in the time spent on the Rotarod (X-axis for both graphs, P < 0.0005). (**E**) For individual mice, 4-AP treatment significantly reduced the average CV of recorded Purkinje cells (red) from untreated (orange) SCA6^84Q/84Q^ mice (P = 0.011 for CV), while (**F**), there was no significant difference on an animal-by-animal basis for Purkinje cell firing frequency (P = 0.75 for frequency). For (**E**,**F**), open circles show data for individual mice, while filled circle show averages. N = 5–8 Purkinje cells/animal for N = 3 vehicle-treated and N = 3 4-AP-treated SCA6^84Q/84Q^ mice; Comparisons made with one-way ANOVA followed by post-hoc Tukey test for (**C**,**D**), and Student’s t test for (**E**,**F**); *P < 0.05; **P < 0.01; ***P < 0.005.

Since acute *in vitro* administration of 4-AP improves firing precision in Purkinje cells (Fig. 3), we further hypothesized that chronic *in vivo* administration of 4-AP improved motor function in SCA6^84Q/84Q^ mice through a similar mechanism. We made recordings from Purkinje cells in cerebellar slices from 10-month-old SCA6^84Q/84Q^ mice that had chronic 4-AP treatment (for 3 months). Slices were prepared and recorded in the absence of 4-AP in the extracellular solution (Fig. 5A). We found that Purkinje cells from chronic 4-AP-treated SCA6^84Q/84Q^ mice had significantly improved firing precision compared to chronic vehicle-treated SCA6^84Q/84Q^ mice, as reflected by lower inter-spike interval CV (P < 0.01; Fig. 5B,C), to a level that was indistinguishable from age-matched WT mice (P = 0.52; Fig. 5B,C). However, firing rates in 4-AP-treated SCA6^84Q/84Q^ mice were significantly lower than WT mice (P = 0.024; Fig. 5B,D), and were unchanged from untreated SCA6^84Q/84Q^ mice (P = 0.99; Fig. 5B,D), suggesting that 4-AP rescues motor coordination via its action on Purkinje cell firing precision, not firing rate. Importantly, we found a significant reduction of average Purkinje cell spike precision (averaging 5–8 cells/animal) in each 4-AP-treated mouse compared to control SCA6^84Q/84Q^ mice (Fig. 5E), and no significant difference was observed for Purkinje cell firing frequency within individual mice (Fig. 5F). These results suggest that mice with more regular Purkinje cell firing have better motor coordination.

**Figure 6:**
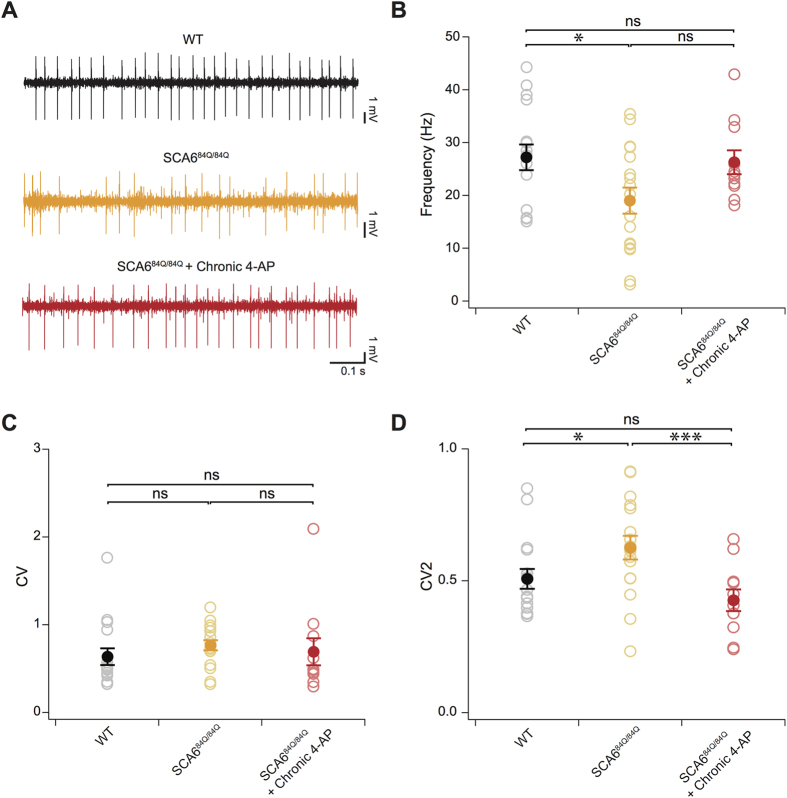
Chronic 4-AP restores *in vivo* Purkinje cell firing precision in SCA6^84Q/84Q^ mice. (**A**) Sample recordings from age-matched WT (top, black trace), vehicle-treated SCA6^84Q/84Q^ (middle, orange trace), and chronic 4-AP-treated SCA6^84Q/84Q^ mice (bottom, red trace) showing the firing precision lost in SCA6^84Q/84Q^ mice is recovered in 4-AP-treated mice. (**B**) Firing frequency is significantly reduced in SCA6^84Q/84Q^ mice but is unaffected by 4-AP treatment (WT: frequency = 27.2 ± 2.4 Hz; SCA6^84Q/84Q^ with vehicle: frequency = 19.0 ± 2.5 Hz; SCA6^84Q/84Q^ + 4-AP: frequency = 26.3 ± 2.3 Hz). (**C**) CV does not accurately measure regularity in *in vivo* Purkinje cell spike trains^31^, and no significant changes are observed across genotype and condition with CV (WT: CV = 0.64 ± 0.10; SCA6^84Q/84Q^ with vehicle: CV = 0.77 ± 0.06; SCA6^84Q/84Q^ + 4-AP: CV = 0.69 ± 0.15). (**D**) However, CV2, a better measure of spike regularity in *in vivo* Purkinje cell recordings^31^, reveals that Purkinje cells fire with significantly less firing precision in anaesthetized SCA6^84Q/84Q^ mice and that firing precision is recovered upon chronic 4-AP treatment (WT: CV2 = 0.51 ± 0.04; SCA6^84Q/84Q^ with vehicle: CV2 = 0.63 ± 0.04; SCA6^84Q/84Q^ + 4-AP: CV2 = 0.43 ± 0.04). N = 16 cells for WT; N = 17 cells for SCA6^84Q/84Q^ mice and N = 11 for SCA6^84Q/84Q^ + 4-AP mice. Comparisons made with one-way ANOVA followed by post-hoc Student’s t test for (**B–D**); *P < 0.05; ***P < 0.005.

Finally, Purkinje cells fire more irregular simple spike trains *in vivo* than in acute brain slices (e.g.^30^), raising the question of whether observed changes in firing precision in SCA6^84Q/84Q^ Purkinje cells from acute slices accurately reflects changes in the intact animal during chronic 4-AP administration. To directly address this, single unit recordings were made from Purkinje cells from anaesthetized WT, SCA6^84Q/84Q^, or 4-AP treated SCA6^84Q/84Q^ mice (Fig. 6A). Consistent with our results in slice, we found that firing rates were lower in SCA6^84Q/84Q^ mice than in WT (P = 0.017; Fig. 6B), and that no significant recovery was observed after chronic 4-AP treatment (P = 0.053; Fig. 6B). Quantification of the CV of *in vivo* Purkinje cell firing revealed high values in all conditions, due to the presence of burst firing and variable-length pauses in *in vivo* spike data (Fig. 6C)^31^. Accordingly, we computed each neuron’s CV2, which provides a more meaningful measure of Purkinje cell firing regularity as previously demonstrated^31^,^32^. Firing precision was decreased in SCA6^84Q/84Q^ Purkinje cells, as shown by a significantly increased CV2 compared to WT (P = 0.041; Fig. 6D), while chronic 4-AP administration restored firing precision of SCA6^84Q/84Q^ Purkinje cells to WT levels (comparing CV2 of SCA6^84Q/84Q^ and SCA6^84Q/84Q^ + 4-AP: P = 0.003; CV2 of SCA6^84Q/84Q^ + 4-AP not significantly different from WT, P = 0.21; Fig. 6D). Thus consistent with our findings in acute slice recordings, our *in-vivo* results provide further support that firing precision is reduced in SCA6^84Q/84Q^ mice after 7 months, and that chronic 4-AP administration restores firing precision to WT levels.

## Discussion

Here, we report that Purkinje cell firing precision was reduced in heterozygous SCA6^84Q/+^ mice at an age when motor deficits were detected (19 months), which is particularly relevant for human SCA6 that is typically an autosomal dominant genetic disease. Furthermore, we showed that similar changes in firing precision were observed earlier (i.e., at 7 months) in homozygous SCA6^84Q/84Q^ Purkinje cells, at the time when motor coordination deficits become evident^6^,^10^. These results provide support for the proposal that a reduction in firing precision is an important pathophysiological alteration underlying SCA6. We further found that the precision of action potential timing in homozygous SCA6^84Q/84Q^ Purkinje cells could be rescued acutely with 4-AP, and that chronic administration of 4-AP alleviated ataxic symptoms in SCA6^84Q/84Q^ mice. Motor coordination also improved within a week, continued to improve after 1 month, and then plateaued for as long as we tested, up to 3 months of 4-AP administration. Importantly, this improvement in motor coordination was reversed after 4-AP withdrawal. Purkinje cells showed significantly improved firing precision after chronic 4-AP treatment in SCA6^84Q/84Q^ mice, even in individual mice, and the regularity of 4-AP-treated Purkinje cells was indistinguishable from WT in both acute slice and *in vivo* recordings.

SCA6 is a dominant-negative disease with most patients having a single disease copy of the *CACNA1A* gene^1^. Homozygous mice have often been studied to understand the pathophysiology of SCA6 (e.g.^6^,^7^,^10^), and are more practical to work with since they show motor control deficits at 7 months as opposed to 19 months for heterozygous mice. Indeed, our present findings suggest that homozygous mice are a valuable model system for a largely heterozygous disease. Specifically, at the level of single cells, we found very similar alterations in both firing output in heterozygous and homozygous mice. These parallels support the use of homozygous mice as a model for SCA6, as they suggest that common pathophysiology likely underlies motor coordination deficits regardless of gene dose.

Reliable transmission of information in the cerebellar microcircuit is critical for cerebellar function. Our results provide further evidence of the importance of Purkinje cell firing to this process. Interestingly, several different types of ataxia appear to share a common motif of Purkinje cell firing rate and/or firing precision impairment, including EA2^16^, SCA1^20^,^21^, SCA2^23^,^24^, SCA3^25^, and a different mouse model of SCA6^9^. In this recent SCA6 study, Mark and colleagues overexpress a pathological C-terminus fragment of α1A subunit of P/Q channel associated with SCA6, leading to firing precision abnormalities months after the onset of motor coordination deficits^9^. Together with our findings that firing abnormalities were observed at 7 and 10 months in homozygous SCA6^84Q/84Q^ mice, as well as 19 months in heterozygous SCA6^84Q/+^ mice, these findings argue that Purkinje cell firing deficits may underlie motor coordination deficits over a long time period during when symptoms is observed in SCA6 patients, although we expect that other network deficits^9^, and Purkinje cell death^10^, also contribute as the disease progresses. Firing abnormalities in principal cell types across brain regions have been posited to contribute to several different neurodegenerative diseases^33^, and our study provides further support for this hypothesis.

Given the importance that firing rate and spike timing precision appears to have for cerebellar function from our study and others^16^, it is surprising that Purkinje cell firing rates are less regular *in vivo* (e.g.^30^; Fig. 6) than the *in vitro* slice preparation (Figs 1, 2 and 5). However, although *in vivo* firing appears irregular, close analysis of its structure reveals epochs of regular firing separated by pauses of variable duration, suggesting that even *in vivo*, firing regularity is important in the Purkinje cell signalling^31^. We recorded *in vivo* in anaesthetized mice, which has been shown to result in lower Purkinje cell firing rates than in awake behaving mice^34^, although spike regularity, as measured by both CV and CV2, was not significantly different in anaesthetized and awake mice^34^, suggesting that the changes we observe reflect changes in behaviorally-relevant states. Our *in vivo* results (Fig. 6) confirm that Purkinje cell firing regularity is reduced in SCA6, even accounting for the fact that firing is initially less regular *in vivo* in WT mice.

4-AP is an FDA-approved drug that improves symptoms in patients with MS^26^,^27^ and has been shown to reduce the incidence of ataxic attacks in mouse models of EA2^18^,^19^, SCA1^21^, and in EA2 patients^35^. Although 4-AP did not fully rescue ataxia in our mouse model of SCA6 to WT levels, this may be due to drug dose, since we found a positive correlation between drug intake and motor performance (Fig. 4E). Alternatively, partial rescue may arise because some changes in the SCA6 brain are not ameliorated by 4-AP. Future work will be needed to establish the effects of 4-AP on other brain regions.

4-AP blocks voltage-dependent K^+^ channels like K_v_1.5 and others^18^, and may improve spike precision in SCA6^84Q/84Q^ mice through an enhancement of the action potential afterhyperpolarization^18^, Although the precise mechanism of action of 4-AP is not fully understood, it has been shown to selectively restore firing precision^18^ and firing rate^21^ in other ataxia mouse models, suggesting that the action of 4-AP on Purkinje cell firing depends on the underlying pathological changes that may differ in different ataxias. It will be particularly interesting to understand why average firing rate abnormalities are rescued in other ataxias, like SCA1^21^, but not in our study with SCA6^84Q/84Q^ mice or in EA2 mice^18^. However, both SCA6 and EA2 involve mutations in the α1A subunit of the P/Q channel, and clinical overlap has suggested that these two disorders may be a part of a complex disease with variable symptoms^1^. Our findings that firing precision alterations in SCA6 mice can be rescued by chronic 4-AP treatment are similar to those of studies on EA2 mutant mice, by Khodakhah and colleagues^16^,^18^,^19^, supporting the hypothesis of overlapping pathophysiology for SCA6 and EA2. Although a direct link between the SCA6 mutation and a 4-AP-sensitive K^+^ channel has not yet been uncovered, the pathway may be complex given that SCA6 likely alters the function of the α1ACT transcription factor^8^ in addition to its effects on P/Q channels.

Firing abnormalities and ataxia have been ameliorated by other approaches in different forms of ataxia, which should be investigated in SCA6 in future studies. For example, a similar drug to 4-AP, 3,4-diaminopyridine (DAP), has been shown to improve other motor symptoms, like downbeat nystagmus, in human ataxias including SCA6^36^, and also improves Purkinje cell firing rate and motor coordination deficits in SCA1 mice^21^, making DAP an interesting drug to study in SCA6 as well. Other approaches, such as modulating or enhancing K^+^ channel function pharmacologically^16^,^18^,^19^,^24^ or via viral expression of K^+^ channels^22^ can improve motor symptoms in other forms of ataxia such as EA2^16^,^18^,^19^, SCA2^24^, and SCA1^21^,^22^, and should be studied further for SCA6.

Our findings suggest 4-AP as a pharmacological treatment for SCA6, a devastating form of ataxia that currently has no known cure or treatment. As 4-AP is already approved for use in people, our findings support the NIH strategy of drug repurposing^37^ that is of particular relevance for rare diseases such as SCA6, for which drug companies may be reluctant to invest in novel drug development due to the small market potential. Our results show that long-term partial rescue of motor deficiencies is possible in SCA6. Further studies are required to determine whether disease symptoms can be fully rescued in SCA6 patients with this pharmacological approach. Finally, our results contribute to the body of evidence arising from multiple ataxia mouse models suggesting that Purkinje cell firing deficiencies are one of the contributing factor for motor coordination deficits, which suggests that treatments for rare forms of ataxia that have not yet been studied with animal models may nonetheless share similar mechanisms and therefore treatment strategies.

## Methods

### Animals

Transgenic mice harboring a humanized 84-CAG repeat tract knocked into the *CACNA1A* locus was obtained from Jackson laboratories (Bar Harbor, Maine; strain: B6.129S7-Cacna1a^tm3Hzo^/J; stock number: 008683), and heterozygous mice were bred in our animal facility to obtain litter-matched male and female homozygous (SCA6^84Q/84Q^), heterozygous (SCA6^84Q/+^), and wildtype (WT) control mice that were used in the experiments. Animal procedures were approved by the McGill Animal Care Committee and conform to the guidelines set in place by the Canadian Council on Animal Care.

### Behavior

We use a Rotarod (Stoelting Europe, Ireland) as previously described^10^ to assay motor coordination deficits in SCA6^84Q/84Q^ mice since we found in a previous study that changes identified with Rotarod were consistent with other motor assays such as the elevated beam and swimming assay, but that results with Rotarod were more robust^10^. After one hour of acclimatization in the experimental room, mice were placed on an accelerating rotarod (each trial could last up to 10 minutes included an initial 5 minute period during which the rod is slowly accelerated from 4 to 40 RPM, followed by another 5 minutes with a constant speed of 40 RPM, although mice typically fell off before the end of the trial), and the latency to fall was recorded. Heterozygous SCA6^84Q/+^ mice were run for 4 Rotarod trials/day over 4 days; results from day 4 are shown in Fig. 1E. For homozygous SCA6^84Q/84Q^ mice, 4 trial/day was conducted from day (D) 0–14, followed in some mice by further Rotarod testing (in 4 or 5 day blocks; 4 trials per day) at 1 and 3 months.

### Acute cerebellar slice preparation

Mice were anaesthetized using intraperitonial injection of Avertin (2,2,2-tribromoethanol; dosage: 0.2 mL/10 g body weight), after which rapid transcardial perfusion with ice-cold partial sucrose replacement solution ((in mM) 111 Sucrose, 50 NaCl, 2.5 KCl, 0.65 CaCl_2_, 10 MgCl_2_, 1.25 NaH_2_PO_4_, 25 NaHCO_3_ and 25 glucose, bubbled with 95% O_2_ and 5% CO_2_ to maintain pH at 7.3; osmolality 320 mOsm) using a peristaltic pump (Gilson) for ~1.5–2 minutes was performed to ensure good slice quality. Mice were then decapitated and their brains were extracted and sliced in ice-cold partial sucrose replacement solution (composition same as above except for containing 0.5 mM CaCl_2_). 250 μm-thick parasagittal cerebellar vermis slices were cut using a Leica VT 1000S Vibratome and were incubated in artificial cerebrospinal fluid (ACSF; (in mM) 125 NaCl, 2.5 KCl, 2 CaCl_2_, 1 MgCl_2_, 1.25 NaH_2_PO_4_, 26 NaHCO_3_ and 20 glucose, bubbled with 95% O_2_ and 5% CO_2_ to maintain pH at 7.3; osmolality 323 mOsm) at 37 °C for 45 min before conducting electrophysiology experiments^14^.

### Slice Electrophysiology

Extracellular recordings were made from visually identified soma of Purkinje cells in slices with an upright microscope (Scientifica, UK). The slices were bathed with ACSF at 33–35 °C for up to 5 hours. Pipettes (1–2 μm tip) were filled with ACSF and 1–3 simultaneous recordings were acquired with an Axopatch 700B patch-clamp amplifier (Axon Instruments, Foster City, CA). Custom data acquisition and analysis software was employed using IGOR Pro software (Wavemetrics, Portland, OR) to determine the timing of action potentials, and the mean firing rate and coefficient of variation (CV) of inter-spike interval of simple spikes were calculated to determine the average firing regularity. For pharmacological application, drugs were bath perfused in the ACSF after recordings were established. A cocktail of synaptic blockers containing DNQX (10 μM), APV (50 μM), and SR95531 (10 μM), was bath applied to slices to block fast excitatory and inhibitory synaptic input to Purkinje cells where indicated (Supplementary Fig. 1), and 4-aminopyridine (5 μM) was applied either with the synaptic blocker cocktail or on its own (Fig. 3, Supplementary Fig. 1). All chemicals were purchased from Sigma-Aldrich (Oakville, ON, Canada) unless otherwise noted.

### *In Vivo* Surgery and Recording

*Surgical preparation*. Prior to surgery, each mouse received an analgesic subcutaneous injection of carprofen (4 mg/ml). Mice were then anesthetized with an intraperitoneal injection of ketamine (10^−1^ mg/g), acepromazine maleate (3 × 10^−2^ mg/g), xylazine (10^−2^ mg/g) and sterile saline, and were then fixed in a stereotaxic frame. An incision was made to expose the interparietal and occipital area of the skull. A craniotomy was performed to reveal the cerebellum, and care was taken to maintain a stable plane of anaesthesia and prevent dehydration or hypothermia throughout the experimental procedures.

#### In Vivo Single-Unit Recording Data Acquisition

Extracellular single-unit activity was recorded using glass electrodes (Sutter Instrument) filled with 3M NaCl (8–12 MΩ of impedance). The electrode was inserted into the cerebellum and gradually advanced by a microdrive (Narishige International, Amityville, NY) into the anterior lobules of the cerebellum. Single-unit activity was attained using a Plexon system and the signal were band-pass filtered at 400–5000 Hz and sampled at 20 kHz. After each recording session, the mouse was sacrificed, and the recording glass electrode was removed and replaced with a tungsten electrode. Electrolytic lesions were then made using a lesion producing device (Stoelting, Kiel, WI), and the brain was then extracted and cut in sagittal section to identify the recording sites.

#### In Vivo Single-Unit Recording Data Analysis

Raw spike signals were imported into MATLAB (The MathWorks, Natick, MA) and analyzed using custom written algorithms. Purkinje cells were first identified based on the occurrence of both simple spikes and complex spikes. The spike timing precision was quantified using CV. CV is calculated using the formula, 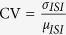, where σ and μ are the standard deviation and mean of the inter-spike intervals (ISI). In addition to CV and frequency of simple spikes, the CV2, which measures short-scale regularity in spike trains^32^, was also calculated where 
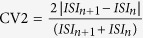
. All *in vivo* data were acquired and analyzed blind to condition.

### *In vivo* Drug Administration

Because 4-AP has a short half-life in rodents of ~2 h, oral administration via drinking water is a reasonable means of maintaining a relatively constant plasma concentration during their waking cycle^18^, since water consumption follows a circadian rhythm in mice, with elevated and relatively constant levels throughout the waking cycle^38^. Mice were housed singly and were provided with 425 μM 4-AP in a 10% sucrose drinking water solution (to make solution palatable), made up fresh daily from a frozen 4-AP stock, which is estimated to maintain a plasma concentration of 4-AP of ~0.5 ng/ml, within the range of concentrations measured in patients treated for MS with a sustained-release oral administration^26^,^27^. Water consumption was measured daily and found to be ~10 ml/day, which is broadly similar to water consumption in previous studies^38^, although higher than a recent 4-AP study where mice consumed roughly 2.5 ml/day^18^, possibly because mice were older in our study. Control mice were provided with 10% sucrose drinking water without drug. Mice were provided with 4-AP for at least 7 days and up to 3 months, and animals underwent Rotarod behavioral testing as described above. Mice were monitored daily and weighed every other day, and 4-AP treatment was not found to have any effects on body weight compared to WT (Supplementary Fig. 2; ANOVA fit model, F_27,330_ = 0.7209, P = 0.847). To look for a correlation between drug intake and motor performance, we used 1 week without drug (1 week – 4-AP) instead of day 0 (pre-drug) in Fig. 4E,F to control for motor learning that occurs during the first week in all conditions. A significant correlation was observed for SCA6^84Q/84Q^ mice in Fig. 4E even when 1 week – 4-AP data was removed (*R* = 0.54; P = 0.0011).

### Statistics

Comparisons were made using either one-way ANOVA followed by Tukey’s HSD or Student’s post-hoc t-test using JMP software (SAS, Cary, NC), or paired or unpaired two-tailed Student’s t tests assuming unequal variance using Igor Pro software, with Bonferroni correction for multiple comparisons. Data are reported as Mean ± S.E.M.

## Additional Information

**How to cite this article**: Jayabal, S. *et al*. 4-aminopyridine reverses ataxia and cerebellar firing deficiency in a mouse model of spinocerebellar ataxia type 6. *Sci. Rep*. **6**, 29489; doi: 10.1038/srep29489 (2016).

## Supplementary Material

Supplementary Information
