# Supplementary material for: 4-aminopyridine reverses ataxia and cerebellar firing deficiency in a mouse model of spinocerebellar ataxia type 6
Source: Sci Rep. 2016 Jul 6;6:29489. doi: 10.1038/srep29489 (PMC4933933; doi:10.1038/srep29489)
Supplement: Supplementary Information [file srep29489-s1.pdf]

4-aminopyridine reverses ataxia and cerebellar firing deficiency in a mouse model of spinocerebellar ataxia type 6

Sriram Jayabal[1,2], Hui Ho Vanessa Chang[3], Kathleen E. Cullen[3], and Alanna J. Watt[1,*]

Supplementary Information

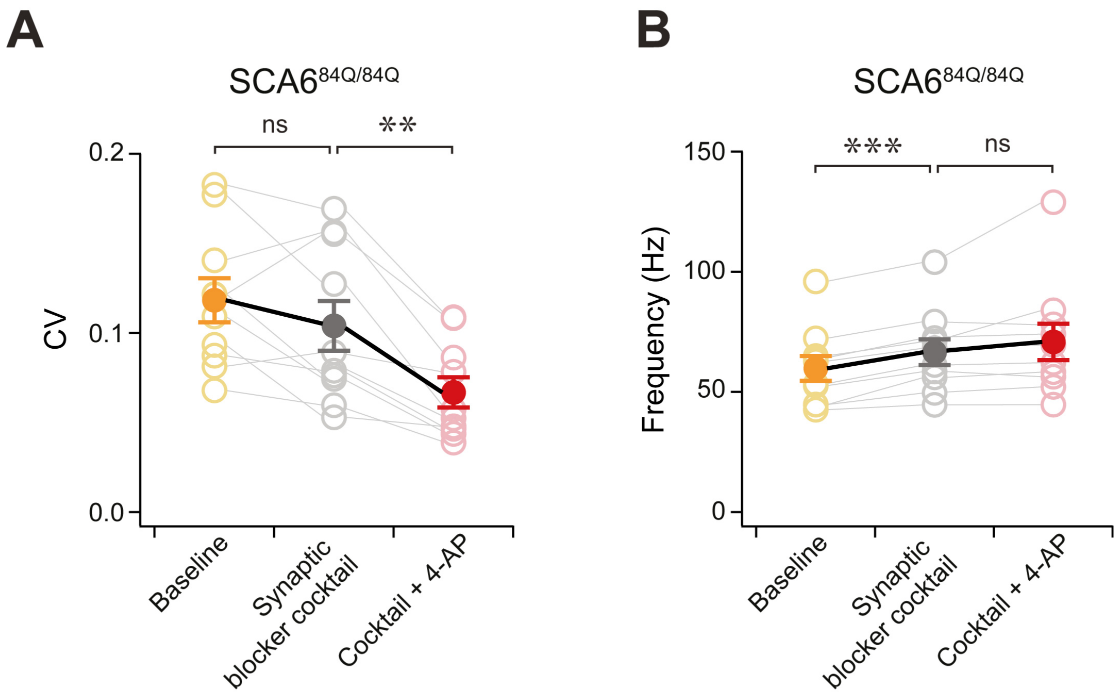

Supplementary Figure 1. 4-AP does not act on presynaptic currents to increase Purkinje cell firing precision. (**A**) A cocktail of synaptic blockers of fast excitatory (AMPA, NMDA) and inhibitory ($GABA_A$) transmission did not alter firing precision (as measured by CV, $P = 0.14$), while the further addition of 4-AP increased the regularity of Purkinje cell firing ($P = 0.0008$; $N = 10$ Purkinje cells from $N = 3$ $SCA6^{84Q/84Q}$ mice; paired Student's t test, Bonferroni corrected, significance level $\alpha = 0.025$). (**B**) Synaptic blockade increased firing rate in 7-month-old $SCA6^{84Q/84Q}$ mice (grey markers, $P < 0.0001$), which is not altered after the subsequent application of synaptic blocker cocktail + 4-AP (red markers, $P = 0.14$). Paired Student's t test, Bonferroni corrected, significance level $\alpha = 0.025$. ** $P < 0.001$; *** $P < 0.0001$.

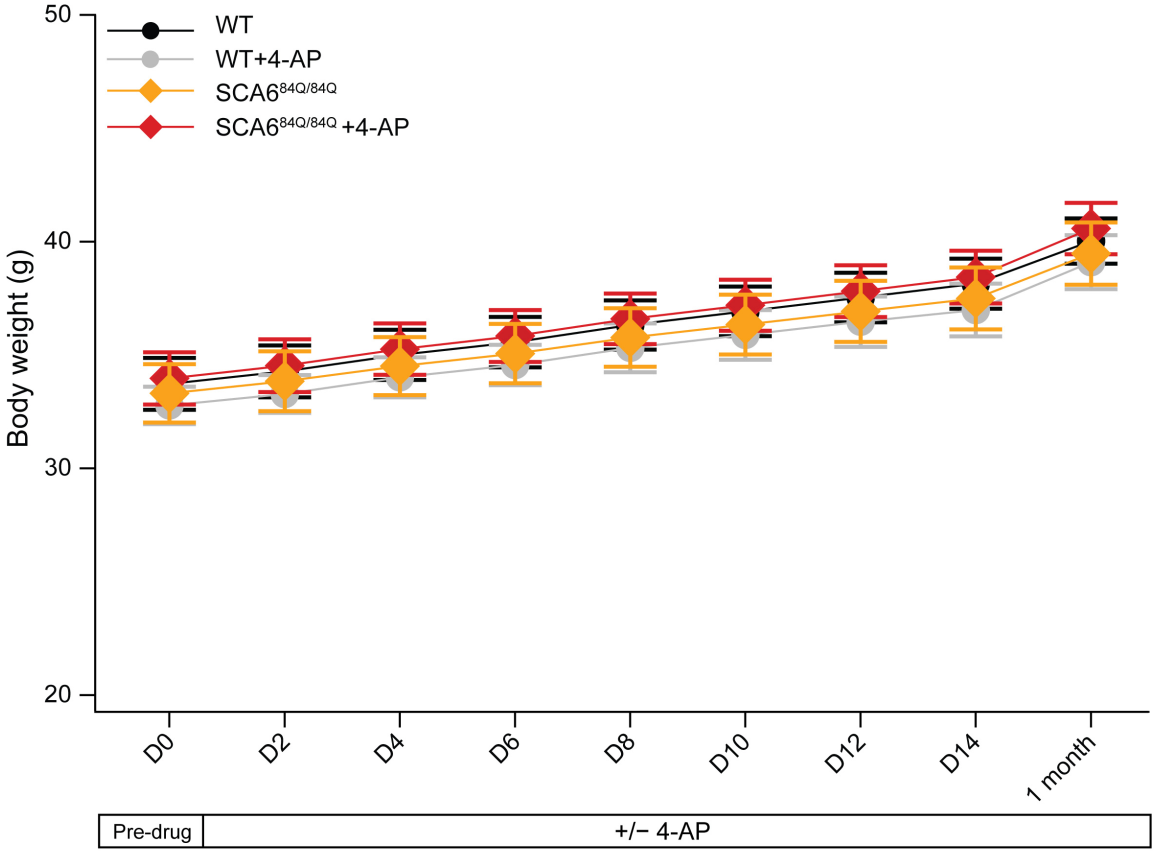

Supplementary Figure 2. 4-AP administration does not affect weight of mice. Mice were weighed on alternating days as a means of assessing their health either with 4-AP in their drinking water or without. Weight was indistinguishable across genotype and condition at all days measured (WT with vehicle: unfilled circles, N = 11; WT + 4-AP: black circles, N = 11; SCA6$^{84Q/84Q}$ with vehicle: orange diamonds, N = 9; SCA6$^{84Q/84Q}$ + 4-AP: red diamonds, N = 9; ANOVA fit model, $F_{27,330} = 0.7209$, $P = 0.847$; not significantly different at 3 months, data not shown, $P = 0.36$).
